# Poor outcomes in recurrent tuberculosis: More than just drug resistance?

**DOI:** 10.1371/journal.pone.0215855

**Published:** 2019-05-06

**Authors:** Danielle B. Cohen, Geriant Davies, Wakisa Malwafu, Helen Mangochi, Elizabeth Joekes, Simon Greenwood, Liz Corbett, S. Bertel Squire

**Affiliations:** 1 Malawi Liverpool Wellcome Trust Research Programme, Blantyre, Malawi; 2 Department of Clinical Sciences, Liverpool School of Tropical Medicine, Liverpool, United Kingdom; 3 Department of Infection, Immunity & Cardiovascular Disease, University of Sheffield, Sheffield, United Kingdom; 4 Institute of Global Health, University of Liverpool, Liverpool, United Kingdom; 5 Malawi College of Medicine, University of Malawi, Blantyre, Malawi; 6 Department of Radiology, Royal Liverpool and Broadgreen University Hospitals Trust, Liverpool, United Kingdom; 7 Department of Clinical Research, London School of Hygiene and Tropical Medicine, London, United Kingdom; Jamia Hamdard, INDIA

## Abstract

**Background:**

Approximately 11% of people reported to have tuberculosis (TB) have previously received treatment. Clinical outcomes are consistently poor on retreatment regimens, however reasons for this are unclear. This study aimed to explore factors which may contribute to unsuccessful outcomes in retreatment TB.

**Methods and findings:**

A prospective cohort of consecutive patients starting WHO Category II retreatment regimen was recruited at a central hospital in Malawi. Participants were evaluated at baseline, after completion of the intensive phase at 2-months, and at the end of the 8-month treatment course. Patients were assessed for respiratory co-morbidity; anaemia; renal impairment; diabetes; Anti-retroviral (ART) failure; and drug toxicity. Amongst 158 patients entering TB care at the point of a recurrent episode, only 92 (58%) had a microbiologically confirmed diagnosis. The prevalence of drug resistance was low (9.6%). Of the 158 patients, 131 (83%) were HIV-positive, of whom 96 (73%) were on ART. Of 63 patients on ART >1 year, 24 (38%) had ART failure. Chronic lung disease was found in 88% on CT thorax, including scarring (80%), bronchiectasis (61%), COPD (22%), and destroyed lung (19%). Spirometry revealed restrictive deficit in 60%, and obstructive deficit in 7% of patients. Anaemia and renal impairment were common (34% and 45% respectively). Ototoxicity developed in 32%, and nephrotoxicity in 15%. 40% of patients reported peripheral neuropathy. Liver injury developed in 4%.

**Conclusions:**

If outcomes are to be improved in retreatment TB, there is an urgent need to address the impact of other co-morbid medical conditions including chronic lung disease, HIV and ART failure.

## Introduction

Each year, approximately 11% of all people reported to have tuberculosis (TB) have previously received treatment, which in 2016 amounted to 0.6 million people [1]. Although it accounts for a minority of those treated for TB, retreatment TB is associated with a disproportionate burden for patients, families and health systems.

Patients with ‘retreatment TB’ form a heterogeneous group of people who have previously been treated for TB. They comprise those with microbiologically confirmed relapse after successful treatment; treatment failure (defined as remaining smear or culture positive after 5 months of treatment); treatment following loss to follow-up; and people classified as ‘other’ who have had previous treatment but do not fulfil any of the other definitions [2]. In 1991, The World Health Organization (WHO) recommended that people presenting with TB in settings with a low prevalence of multidrug-resistant TB (MDR-TB) who have previously been treated were prescribed a standardised regimen consisting of Rifampicin(R), Isoniazid(H), Pyrazinamide(Z), Ethambutol(E) and Streptomycin(S) for 2 months, followed by RHZE for 1 month and then RHE for 5 months [3]. This ‘Category II’ regimen has no clinical trial evidence base, and treatment outcomes in this group of patients are persistently reported to be poor [4–7]. The most recent WHO TB treatment guidelines therefore recommend discontinuation of the Category II regimen where possible in favour of regimens which are guided by the results of drug susceptibility testing (DST) [8].

Whilst it is often assumed that poor outcomes are due to the increased prevalence of drug resistance in this group, rates of successful treatment are sub-optimal even in regions where the prevalence of drug resistance is low and when those with rifampicin resistance are treated with second line agents [1]. A study in Zimbabwe suggested that there is a significant burden of chronic lung disease in patients on TB retreatment, and that empirical treatment for TB may be masking a bigger problem of chronic lung disease [9], but no research has comprehensively addressed the reasons for poor treatment outcomes in patients accessing care at the point of a second TB episode.

We conducted a prospective study in Malawi of patients presenting with TB who had previously received treatment. The aim of the study was to evaluate the problem of poor outcomes in patients with retreatment TB by examining the burdens of drug resistance, medical co-morbidity and drug toxicity in patients prescribed TB retreatment.

## Methods

### Study design and participants

Queen Elizabeth Central Hospital is the largest hospital in Malawi, treating approximately 2000 patients for tuberculosis each year. All patients with retreatment tuberculosis in the Blantyre district are referred for management. At the time of the study, all retreatment patients in Malawi were prescribed the standard WHO category II regimen. A prospective descriptive study was conducted in which consecutive adult patients being registered with retreatment tuberculosis over a 15-month time period were recruited. The rate of successful treatment for ‘new’ (non-retreatment) patients in Malawi during the period of the study was 85% [10].

Patients were included in the study if they were ≥16 years old and able to provide informed consent. Patients were excluded if they had MDR-TB identified on drug sensitivity testing or rifampicin resistance detected using molecular techniques. All participants provided written (or, in the case of those who were illiterate, witnessed) informed consent. Ethical approval for the study was obtained from The Malawi College of Medicine and The Liverpool School of Tropical Medicine.

### Procedures

Patients were assessed during routine TB follow-up visits at baseline, two months and completion of treatment.

#### Laboratory procedures

Laboratory tests were carried out at the Malawi-Liverpool-Wellcome Research Programme, Malawi College of Medicine (Blantyre) or University of North Carolina laboratory (Lilongwe). Serum creatinine, ALT and bilirubin were measured using Beckman Coulter Synchron CX5 PRO Clinical System. Kidney injury was defined as increase in creatinine of 1.5-fold, or reduction in CrCl by ≥25%. Renal impairment was defined as creatinine >115μmol/L or CrCl <90 mL/min. Drug induced liver injury was diagnosed if ALT >200 IU/L; rise in bilirubin to >40 μmol/L; or clinical jaundice [11–13]. Full blood count was performed using Beckman Coulter HmX analyser. Anaemia was defined as Hb <8g/dL [14]. HbA1c was performed using AU480 Beckman Coulter analyser. Diabetes was diagnosed if HbA1c ≥6.5% [15].

In HIV-positive patients, CD4 count was performed using CyFlow Counter (Sysmex PartecGmbH, Gorlitz, Germany). HIV viral load (VL) was performed using Abbott m2000 RealTime System (Illinois, USA) in patients who had been on ART > one year. ART failure was diagnosed using the standard operational definition in Malawi (HIV VL >5000 copies/ml).

Sputum samples were stained with Auramine-O and examined using florescence microscopy. Liquid culture was performed using BACTEC MGIT 960 Mycobacterial Detection System (Becton, Dickinson and Company, New Jersey, USA). XpertMTB/RIF assay (Cepheid Sunnyvale, USA) was performed on unprocessed sputum samples according to manufacturer’s instructions. Microbiologically confirmed TB was defined as sputum smear, culture or XpertMTB/RIF positive. Successful treatment was defined as a combination of ‘cured’ and ‘treatment completed’ [2].

#### Radiology

Chest radiographs were performed at the start of treatment at the QECH department of radiology. In a subgroup, Computerised Tomography (CT) thorax was performed during the first two months of treatment. The CT scanner in Blantyre was not functional at the start of the study, and CT was performed on consecutive patients once it was available. CT was performed on a SOMATON Sensation 16 slice scanner (Siemens) using the pre-programmed, low dose, unenhanced lung protocol. Images were reviewed using OsiriX-HD software. Two radiologists independently reported all images, followed by a consensus approach to resolve discrepancies on final diagnosis. All reports were recorded on standardised proformae developed from established guidelines [16].

#### Audiology

Audiological assessments were done at baseline, 2 months and 8 months. Otoscopy was performed to exclude abnormality in the auditory canal or tympanic membrane. Pure Tone Audiometry (PTA) was performed using the KUDUwave 5000 audiometer (eMoyoDotnet (Pty) Ltd, Randburg, South Africa). PTA thresholds were obtained at frequencies of 250–1600 kHz. In line with guidelines [17–18] significant change in hearing was defined as ≥20 dB change from baseline at any one test frequency; or ≥10 dB change from baseline at two consecutive frequencies. To improve specificity for ototoxicity, this definition was refined to include only change in hearing at high frequency (≥8000 kHz). Vestibular assessment included Romberg’s test, head thrust test, and clinical test of sensory integration of balance.

#### Lung function

Spirometry was performed on patients at 2 and 8 months using the EasyOne spirometer (ndd Medical Technologies, MA, USA), according to manufacturer’s instructions. Spirometry was performed at 2-months rather than at baseline in order to obtain an accurate evaluation of underlying lung function after resolution of the acute episode; and because of infection control concerns. Results were compared to predicted levels using NHANES III reference ranges. For quality control, a second reader reviewed 20% of all tests. Interpretation of spirographs was based on the GOLD criteria [19].

### Statistical analysis

This was a descriptive study recruiting consecutive patients over a defined time period and therefore not based on sample size calculation. Exploratory analyses were performed to evaluate possible associations with clinical outcome using multivariate logistic regression models with predefined variables.

## Results

**Fig 1:**
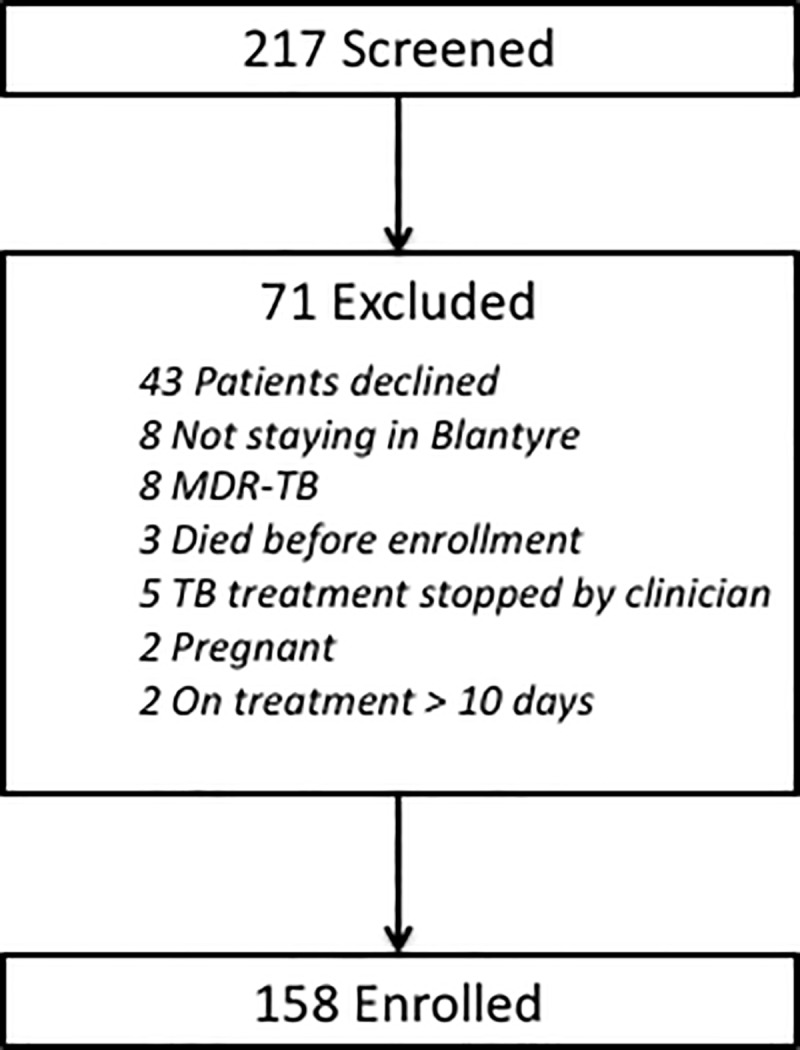
Study flow diagram.

Between June 2013 and September 2014, 158 patients were enrolled (Fig 1). Baseline characteristics of the study population are shown in Table 1.

**Table 1:** Baseline characteristics of study participants.

	Total study population n = 158
Patient age (median, IQR)	37 (31–43)
Patient sex (% male)	102 (64·6)
TB Class	
Pulmonary	132 (83·5)
Extra-pulmonary	26 (16·5)
TB Category *	
Relapse	62 (39·2)
TALTF	7 (4·4)
Fail	5 (3·2)
Other	84 (53·2)
No. of previous TB episodes	
1	138 (87·3)
2	17 (10·8)
>2	3 (1·9)
HIV status	
Negative	27 (17·1)
Positive	131 (82·9)
CD 4 count (median, IQR)	171 (61–324)
ART failure (n = 64)	24 (37·5)
Karnofsky score at baseline (median, IQR)	90 (80–100)
HR >100bpm	95 (60·1)
RR>20bpm	37 (23·4)
SBP<100mmHg	50 (31·7)
BMI	
≥18.5	36 (23·7)
16–18.4	75 (49·3)
<16	41 (27·0)
Anaemia	34 (22·2)
Diabetes	5 (3·3)
Renal impairment	52 (33·8)
Chronic Lung disease on CT Thorax (n = 102)	90 (88·2)
Pulmonary function testing (n = 103)	
Normal	34 (33·0)
Obstructive	6 (5·8)
Restrictive	62 (61·2)
Smoker	
Never	116 (73·4)
Former	41 (26·0)
Current	1 (0·6)
History of alcohol excess	27 (17·1)

* Relapse = a patient previously treated for TB, declared cured or treatment completed, who is diagnosed again with smear or culture positive TB; Treatment after failure = a patient who is started on retreatment regimen after having failed previous treatment; Treatment after loss to follow up (TALTF) = a patients who returns to treatment, positive bacteriologically, following interruption of treatment for 2 or more consecutive months; Other previously treated = all previously treated cases that do not fit any of the above definitions http://www.who.int/tb/err/rr_final_forms_en.pdf.

### Tuberculosis factors

Only 92/158 (58%) had a microbiologically confirmed diagnosis and most (53.2%) of patients were classified as ‘other’. There were no significant associations between microbiologically confirmed TB and age, gender, HIV, chronic lung disease or diabetes. From patients who were sputum culture positive, fifty-two isolates were recovered for phenotypic drug sensitivity testing. There were no significant associations between having had DST performed and gender, HIV status, the presence of chronic lung disease or abnormal spirometry. After screening for rifampicin resistance with GeneXpert, there was no undiagnosed rifampicin resistance. Sputum from only five patients (9.6%) cultured *M*. *tuberculosis* isolates with drug resistance (3 ethambutol resistance; 2 isoniazid resistance).

### HIV factors

HIV prevalence was 82.9% (compared to 56% HIV prevalence reported in ‘new’ TB patients in Malawi at the time of the study [20]); and the median CD4 count of HIV-positive patients was 171 cells/mm^3^. There were 63 patients who had been on ART >1 year, and 24 (37.5%) of these had ART failure.

### Other co-morbidities

Whilst baseline anaemia and renal impairment were common (22.2% and 33.8% respectively), diabetes was present in only 3.3% of patients. Of 102 patients who had CT thorax performed, 88 (86.3%) had chronic lung disease. Bronchiectasis was present in 62 patients; features of emphysema in 22; scarring in 20; and destroyed lung in 19. There were no significant differences in the rates of chronic lung disease between those patients who were sputum culture positive and those who were sputum culture negative. The majority (71%) of bronchiectasis was cystic bronchiectasis. Patients with bronchiectasis on CT were more likely to have changes consistent with acute pulmonary disease on chest x-ray than those without bronchiectasis (95.2% v 71.1%, p-value 0.002). Of 62 patients with bronchiectasis on CT thorax, 32 (52%) had no evidence of bronchiectasis on plain chest x-ray. After adjusting for age, gender, smoking, HIV and confirmed TB, bronchiectasis was associated with being HIV-negative, having confirmed TB and with a history of smoking.

Of the 103 patients who had spirometry performed after 2 months of TB treatment, only 34 (33.0%) had normal pulmonary function: 63 (61.2%) had restrictive deficit and 6 (5.8%) had obstructive deficit. At 8 months, out of 33 patients who had restrictive pulmonary function at 2 months, 19 (57.6%) still had restrictive deficit; 4 had developed evidence of obstructive disease; and 10 had normal spirometry. Obstructive pulmonary function remained at 8 months in 3/4 who had had obstruction at 2 months. Of 21 patients who had normal spirometry initially, 2 developed obstruction and 1 developed restriction.

### Drug toxicity

Overall 80/158 (50.6%) patients experienced at least one drug side effect during the first 2 months of retreatment, with streptomycin toxicity being common: ototoxicity developed in 35.9% and nephrotoxicity in 14.6%. Six patients (4.4%) developed drug induced liver injury, and 51 (40.2%) reported symptoms of peripheral neuropathy.

### Associations with clinical outcome on TB retreatment

The overall rate of successful treatment outcome in the cohort was 68.4%. Mortality was high at 24.1%. In univariate analysis, baseline anaemia and systolic hypotension were both significantly associated with poor end-of-treatment outcome (Table 2). After adjusting for age, gender, HIV status, confirmed TB, hypotension, and anaemia, hypotension but not anaemia remained significantly associated with outcome. Outcomes were worse in the categories ‘Fail’ and ‘Other’, but this difference was not statistically significant.

**Table 2:** Features associated with clinical outcome on TB retreatment.

	Successful outcome n (%)	p-value	OR (95% CI)	p-value	aOR (95% CI)*
Confirmed TB					
No	47/66 (71.2	0.51	0.80 (0.40–1.58)	0.55	0.80 (0.37–1.70)
Yes	61/92 (66.3)				
TB si					
Pulmonary	93/132 (70.5)	0.20	0.57 (0.23–1.36)	-	-
Extra-pulmonary	15/26 (57.7)				
TB Category					
Relapse	47/63 (74.6)				
TLTFU	5/7 (71.4)	0.86	0.85 (0.15–4.83)	-	-
Fail	3/5 (60.0)	0.48	0.51 (0.08–3.34)	-	-
Other	53/83 (63.9)	0.17	0.60 (0.29–1.24)	-	-
Age					
<3	22/32 (68.8)				
30–35	28/43 (65.1)	0.74	0.85 (0.32–2.25)	0.92	1.06 (0.36–3.11)
36–4	39/56 (69.6)	0.93	1.04 (0.41–2.67)	0.74	1.20 (0.40–3.57)
>45	19/27 (70.4)	0.89	1.08 (0.35–3.29)	0.85	1.12 (0.34–3.66)
Gender					
Female	34/56 (60.7)	0.13	1.71 (0.86–3.41)	0.32	1.48 (0.69–3.20)
Male	74/102 (72.6)				
HIV status					
Negative	21/27 (77.8)	0.25	0.56 (0.21–1.50)	0.60	0.74 (0.24–2.30)
Positive	87/131 (66.4)				
HR >100bp					
No	47/63 (74.6)	0.17	0.61 (0.30–1.24)	-	-
Yes	61/95 (64.2)				
RR>20bpm No Yes	64/95 (67.4) 44/63 (69.8)	0.74	- 1.12 (0.56–2.23)	-	-
SBP<100mmHg					
No	82/108 (95.9	0.003	0.34 (0.17–0.70)	0.02	0.41 (0.19–0.87)
Yes	26/50 (52.0)				
BMI					
≥18.5	23/36 (63.9)				
16–18.4	58/75 (77.3)	0.14	1.93 (0.81–4.60)	-	-
<16	24/41 (58.5)	0.63	0.80 (0.32–2.00)	-	-
Anaemia					
No	88/119 (74.0)	0.02	0.40 (0.18–0.87)	0.11	0.50 (0.2.17)
Yes	18/34 (52.9)				
Chronic Lung Disease on CT					
No	11/12 (91.7)	0.32	0.34 (0.04–2.80)	-	-
Yes	71/90 (78.9)				
Abnormal Spirometry					
No	32/34 (94.1)	0.10	0.30 (0.06–1.27)	-	-
Yes	56/69 (81.2)				
Diabetes					
No	101/146 (69.2)	0.61	1.78 (0.19–16.4)	-	-
Yes	4/5 (80.0)				
Renal impairment					
No	74/102 (72.5)	0.11	0.56 (0.28–1.13)	-	
Yes	31/52 (59.6)				

* adjusted for age, gender, HIV, confirmed TB, hypotension, anaemia

### M. tuberculosis burden and drug sensitivity pattern

There was no association between infection with a drug-resistant isolate and unsuccessful clinical outcome (OR 0.64, 95%CI 0.10–4.23; p-value 0.64). The median sputum culture time to positivity (TTP) was 7.8 days (IQR 4.9–9.9 days), and there was no significant association between TTP and clinical outcome (chi^2^ test for trend p-value 0.499).

### HIV

Although the association was not statistically significant, it is of note that 66.4% of HIV-positive patients successfully completed treatment compared to 77.8% of HIV-negative patients. Only 55.4% of patients with CD4 count <100 cells/mm^3^ successfully completed treatment (OR 2.14, 95%CI 1.01–4.53; p-value 0.06). Patients with ART failure had only 50.0% chance of successful treatment outcome, in comparison to 70.1% for patients without ART failure (OR 0.43, 95%CI 0.17–1.05; p-value 0.09).

### Chronic lung disease

Table 3 demonstrates the probabilities of successful treatment outcome were non-significantly lower for patients with any chronic lung disease, bronchiectasis, or scarring. Destroyed lung was associated with unsuccessful clinical outcome on univariate analysis and after controlling for age, gender, smoking, HIV and confirmed TB the adjusted OR for treatment success was 0.32 (95%CI 0.09–1.13).

**Table 3:** Chronic lung disease and TB retreatment outcomes.

	Successful outcome n (%)	p-value	OR	p-value	aOR*
Any CLD					
No	11/12 (91.7)	0.32	0.34 (0.04–2.80)	0.24	0.26 (0.03–2.45)
Yes	71/90 (78.9)				
Bronchiectasis					
No	35/40 (0.88)	0.15	0.45 (0.15–1.35)	0.17	0.41 (0.19–1.47)
Yes	47/62 (0.76)				
Emphysema					
No	64/80 (0.80)	0.85	1.13 (0.33–3.79)	0.91	0.93 (0.25–3.42)
Yes	18/22 (0.82)				
Destroyed lung					
No	70/83 (0.84)	0.04	0.32 (0.11–0.96)	0.08	0.32 (0.09–1.13)
Yes	12/19 (0.63)				
Scarring					
No	18/20 (0.90)	0.24	0.40 (0.08–1.86)	0.09	0.23 (0.04–1.28)
Yes	64/82 (0.78)				

* Adjusted for age, gender, smoking, HIV, onfirmed TB

Successful treatment outcome was seen in 52/63 (82.5%) of patients with restrictive deficit on pulmonary function testing, in comparison to 36/40 (90.0%) without restrictive deficit (OR 0.52, 95%CI 0.15–1.78; p-value 0.39). Of 6 patients who had obstructive pulmonary deficit, only 4 (66.7%) had successful treatment outcome compared to those without obstruction in whom 84/97 (86.6%) successfully completed treatment (OR 0.31, 95%CI 0.05–1.86; p-value 0.21).

### Drug toxicity

There were no significant associations between treatment outcome and nephrotoxicity or ototoxicity (Table 4). Four out of six patients with drug induced liver injury died (RR 5.78, 95%CI 2.75–12.10; p-value 0.004).

**Table 4:** Drug toxicity on TB retreatment.

	Total n (%)	Successful outcome without toxicity n (%)	Successful outcome with toxicity n (%)	p-value	OR
Ototoxicity	28/78 (35.9)	43/50 (86.0)	23/28 (82.1)	0.65	0.75 (0.21–2.63)
Nephrotoxicity	19/130 (14.6)	88/111 (79.3)	15/19 (79.0)	0.97	0.98 (0.30–3.24)
Neuropathy	51/127 (40.2)	62/76 (81.6)	40/51 (78.4)	0.66	0.82 (0.34–1.99)
DILI	6/136 (4.4)	104/130 (80.0)	2/6 (33.3)	0.02	0.13 (0.02–6.15)
SJS	1/158 (0.6)	107/157 (68.2)	1/1 (100.0)	1.0	-

DILI = Drug Induced Liver Injury

SJS = Stevens Johnson Syndrome

## Discussion

This study confirms once again that outcomes in patients receiving recurrent courses of TB treatment are poor with only 68.4% of patients successfully completing treatment. Despite no rifampicin resistance in this cohort, outcomes were no better than those reported for the treatment of MDR-TB in many other cohorts [21]. More importantly, these data also provide new insights into reasons which may explain poor outcomes in patients with retreatment tuberculosis.

In contrast to studies of patients presenting with a first TB episode, neither microbiologically confirmed TB nor TB burden (as estimated by TTP) were predictive of clinical outcome [22–23] in patients entering care having previously received TB treatment. The difference in outcomes between those with and without confirmed TB has previously been ascribed primarily to the fact that patients treated empirically for tuberculosis may not have TB, but rather have other conditions (such as bronchiectasis, cancer or *P*. *jerovecii*) which remain undiagnosed and therefore untreated [24]. A possible explanation for the discrepant result in this study is that, compared to new patients, there is a high prevalence of multiple concurrent diagnoses in patients with retreatment TB regardless of whether they have confirmed tuberculosis; and that co-morbid disease is therefore contributing to unsuccessful treatment in all sub-groups of previously treated patients.

Unlike some countries with a high TB incidence, Malawi has a low prevalence of drug resistant tuberculosis [25], which was shown again in this study. The 0.096 prevalence of drug resistance cannot explain why 31.6% of patients did not complete treatment. If neither microbiologically confirmed TB, high mycobacterial burden, nor drug resistance are accounting for poor outcomes on TB retreatment in this setting, it is probable that other, non-TB, factors are.

The prevalence of medical co-morbidities was indeed extremely high, with 82.9% of patients being HIV infected, 88.2% having chronic lung disease on CT thorax, 33.8% having baseline renal impairment, and 22.2% having anaemia. The study was not powered to detect associations between these factors and clinical outcome, however the proportion successfully completing treatment was non-significantly lower for patients with HIV (66.4% v 77.8%); chronic lung disease (78.9% v 91.7%); renal impairment (59.6% v 72.5%); and anaemia (52.9% v 74.0%).

The prevalence of HIV in this cohort with retreatment TB was 82.9%, higher than the reported 56% prevalence in adults with TB in Malawi [20], and the high proportion of patients with ART failure is a major concern. It is possible that ART failure is common in patients on TB retreatment because this is a group in whom adherence to ART and TB drugs is poor, putting them at risk of both ART failure and retreatment TB. However, people established on ART can live for decades but remain at increased risk of TB throughout that time, thereby increasing their chances of having more than one TB episode. As more people are initiated on HIV treatment in settings with high TB transmission, a growing pool of HIV-positive people living for many years on ART, and therefore at risk of both TB retreatment and ART failure, may develop.

Of interest is the very high burden of chronic lung disease in patients on TB retreatment, and the clear suggestion that this contributes to poor outcomes. The fact that spirometry was performed only on patients who survived to 2-months may mean that the prevalence of lung disease may even have been underestimated. Previous literature had gone some way to describe the features of post-tuberculous lung disease, however none has specifically assessed the burden of lung disease in patients entering care at the point of a retreatment episode [26–27]. If the vast majority of patients starting TB retreatment have underlying chronic lung disease, it is probable that simply treating their respiratory disease with anti-tuberculous drugs will not be sufficient.

After adjusting for age, gender and HIV status the only factor associated with unsuccessful treatment outcome was hypotension at presentation. There are a number of explanations for this association including late presentation with advanced disease [28–29], concurrent bacterial sepsis [30] and hypoadrenalism [31].

The incidence of drug toxicity during the first two months of treatment was high, with 50.6% of all patients experiencing at least one drug side effect. The incidence of streptomycin induced toxicity is of particular concern as 35.0% of patients developed ototoxicity and 14.6% developed nephrotoxicity. Whilst these side effects were not associated with outcome, the high rates are likely to be associated with significant morbidity. This cohort provides the first data on the incidence of streptomycin toxicity in an African cohort on Category II regimen, and adds further weight to the move away from routine use of the regimen.

A weakness of the study is that it was not powered to detect associations between outcome and potential explanatory variables. Hence, whilst it was possible to demonstrate high prevalence of co-morbidity and high incidence of toxicity, the study was frequently unable to show these were statistically associated with outcome. A second drawback is that the study did not recruit a control group and instead comparisons are made to published data. Finally, due to practical and ethical considerations, post mortem examinations were not performed, precluding the establishment of exact cause of death for those who died. Nevertheless, the complexity of the clinical issues facing this very heterogeneous group of patients is highlighted, both in terms of prevalence and severity, and it seems unlikely that such a high burden of comorbid disease is not contributing to poor outcomes.

Overall, this study demonstrates that there may be multiple explanations for the poor clinical outcomes consistently seen in patients who register for TB treatment having previously been treated. The WHO post-2015 strategy for tuberculosis has set a target of 95% reduction in TB deaths by 2035. If these targets are to be met and morbidity and mortality associated with retreatment TB reduced, the TB community must adopt a broader approach to the specific problems faced by patients with retreatment TB. Key issues which need to be addressed include the broad clinical management of patients who present with acute severe illness and hypotension; diagnosis and treatment of non-MDR drug resistance; prevention of drug toxicity; and management of medical co-morbidity especially HIV and chronic lung damage.
